# Lactation induces increased IPSC bursting in oxytocinergic neurons

**DOI:** 10.14814/phy2.14047

**Published:** 2019-04-22

**Authors:** Ion R. Popescu, Zafir Buraei, Juhee Haam, Feng‐Ju Weng, Jeffrey G. Tasker

**Affiliations:** ^1^ Department of Cell and Molecular Biology Tulane University New Orleans Louisiana; ^2^ Tulane Brain Institute Tulane University New Orleans Louisiana; ^3^ Present address: Department of Biology Pace University New York City New York; ^4^ Present address: Neurobiology Laboratory National Institute of Environmental Health Sciences Department of Health and Human Services National Institutes of Health Research Triangle Park North Carolina; ^5^ Present address: Department of Brain and Cognitive Science Massachusetts Institute of Technology Boston Massachusetts

**Keywords:** burst, GABA, IPSC, lactation, oxytocin, synchronization

## Abstract

Hypothalamic magnocellular neurosecretory cells (MNCs) undergo dramatic structural reorganization during lactation in female rats that is thought to contribute to the pulsatile secretion of oxytocin critical for milk ejection. MNCs from male rats generate robust bursts of GABAergic synaptic currents, a subset of which are onset‐synchronized between MNC pairs, but the functional role of the IPSC bursts is not known. To determine the physiological relevance of IPSC bursts, we compared MNCs from lactating and non–lactating female rats using whole–cell recordings in brain slices. We recorded a sixfold increase in the incidence of IPSC bursts in oxytocin (OT)‐MNCs from lactating rats compared to non–lactating rats, whereas there was no change in IPSC bursts in vasopressin (VP)‐MNCs. Synchronized bursts of IPSCs were observed in pairs of MNCs in slices from lactating rats. Our data indicate, therefore, that IPSC bursts are upregulated specifically in OT‐MNCs during lactation, and may, therefore, contribute via rebound depolarization to the spike trains in OT neurons that lead to reflex milk ejection.

## Introduction

The MNCs of the hypothalamic supraoptic nucleus (SON) and paraventricular nucleus (PVN) are neurons specialized for the release of the peptide hormones OT and VP into the blood circulation and within the CNS (Brimble and Dyball 1977; Buijs 1978; Sofroniew 1980; Belin et al. 1984; O'Byrne et al. 1986; Knobloch et al. 2012; Herget et al. 2017). MNC activity is controlled largely by glutamatergic and GABAergic synaptic inputs (Li et al. 2007). GABAergic synaptic inputs to MNCs display two unique features whose function is unknown. First, “shared synapses”, consisting of a single GABAergic presynaptic terminal that forms synapses with two MNCs, were discovered in electron micrographs and have been proposed as a novel mechanism for the generation of synchronized inhibitory inputs to pairs of OT‐MNCs (Theodosis et al. 1981; Hatton and Tweedle 1982; Perlmutter et al. 1984; Theodosis and Poulain 1984; Gies and Theodosis 1994). Second, the MNCs receive spontaneous bursts of high‐frequency IPSCs that can be sharply onset‐synchronized in pairs of cells (Andrew, 1987; Popescu et al. 2010; Potapenko et al. 2011). The bursts of IPSCs are abolished by the GABA_A_ receptor antagonist bicuculline (Popescu et al. 2010). Furthermore, the bursts of IPSCs persist, albeit at a lower incidence, during action potential blockade (Popescu et al. 2010). The robustness of the IPSC bursts predicts a strong impact on neuronal activity, but low burst incidence and lack of a known trigger have made elucidation of their functional significance challenging. However, the hypothalamic magnocellular neurosecretory system undergoes pronounced synaptic plasticity during the perinatal period, and state–/cell–specific plasticity of the IPSC bursts would be suggestive of the bursts’ function (Armstrong et al. 2010).

If IPSC bursts contribute to the OT–mediated milk ejection reflex, for example, then their upregulation would be expected during lactation in the OT‐releasing MNCs. During lactation, the suckling stimulus triggers brief, onset–synchronized, high–frequency trains of action potentials in OT‐MNCs of the paraventricular and supraoptic nuclei (Summerlee & Lincoln, 1981). The onsets of the trains of action potentials in OT‐MNCs are approximately synchronized, although the individual action potentials within the trains and the termination of the trains are not (Belin et al. 1984; see also Rossoni et al. 2008; Honda et al. 2014). The synchronized trains of action potentials cause the pulsatile release of OT from the MNC axons in the posterior pituitary, which results in milk ejection from the mammary glands.

GABA actions contribute to the generation of synchronized spike bursts in the SON during suckling. Interestingly, it has been shown that application of GABA_A_ receptor antagonists to the supraoptic nuclei of lactating rats inhibits the milk ejection reflex (Moos 1995; Voisin et al. 1995). The possible involvement of IPSC bursts in the synchronization of OT‐MNC action potential trains during the milk ejection reflex was suggested by the detection of the synchronized onset of the bursts, since IPSPs can trigger rebound spiking in OT‐MNCs (Stern and Armstrong 1995, 1996; Israel et al. 2008). Multiple studies have reported an increase in the number of shared GABA synapses during lactation, which should increase the incidence of synchronized bursts of IPSCs if shared synapses are the structural substrate for the synchronized bursts (Theodosis et al. 1981; Hatton and Tweedle 1982; Theodosis and Poulain 1984; Gies and Theodosis 1994; see also Perlmutter et al. 1984). Furthermore, the number of canonical, “non‐shared” GABA synapses is increased specifically in OT‐MNCs during lactation, which should cause an increase in IPSC burst incidence (Gies and Theodosis 1994).

We hypothesized that if IPSC bursts play a role in the generation of synchronized action potential bursts during the milk ejection reflex, then the bursts should be regulated by lactation. We performed whole–cell patch clamp recordings in acute brain slices to test if the incidence of IPSC bursts and synchronized IPSC bursts is increased in MNCs from lactating compared to non–lactating female rats, and if this plastic change in GABAergic inputs is specific to OT‐MNCs.

## Methods

### Ethical approval

Female Wistar rats (*Rattus norvegicus*) were used following approval by the Tulane University Institutional Animal Care and Use Committee, and the experiments were performed conformant with the USA national guidelines established by the Association for Assessment and Accreditation of Laboratory Animal Care (AAALAC). The investigators understand the ethical principles under which *Physiology Reports* operates, and the research reported herein complies with the animal ethics checklist.

### Origin and source of rats

Experiments were conducted using wild–type Wistar rats purchased from Charles River Laboratories (Wilmington, MA), transgenic Wistar rats expressing a VP‐GFP fusion gene under the control of the VP promoter (Ueta et al. 2005), and transgenic Wistar rats expressing an OT‐RFP fusion protein under the control of the OT promoter (Katoh et al. 2011). VP‐GFP and OT‐RFP transgenic rat breeders were kindly provided by Professor Yoichi Ueta and were bred and raised in the Tulane University animal facility.

Primiparous lactating rats with litters of 5 to 13 pups were used for experiments when the pups were 9–14 days old. Lactating rats were 108 ± 7 days of age (*n* = 17) and virgin female rats were 136 ± 16 days of age (*n* = 19) at the time of sacrifice. The terms “virgin” and “nonlactating” are used interchangeably in this study. The rats were group‐housed in cages containing red plastic cylindrical shelters for environmental enrichment and had free access to food and water. Virgin rats were group‐housed with female rats following weaning. Pregnant dams were single‐housed for several days prior to parturition, and afterwards were housed with their pups.

### Brain slices

Rats were decapitated, under general anesthesia with isoflurane inhalation, and coronal slices, 350 *μ*m in thickness and containing the SON or PVN, were cut on a vibrating microtome (Pella Instruments, Redmond, CA), as described previously (Di et al. 2003). Prior to recordings, the slices were stored for at least 2 h at room temperature in a holding chamber in aCSF containing (in mM): 140 NaCl, 3 KCl, 1.3 MgSO_4_, 1.4 NaH_2_PO_4_, 2.4 CaCl_2_, 11 glucose, and 5 HEPES, which was bubbled with 100% O_2_. The pH of the aCSF was adjusted to 7.2–7.3 with NaOH. For recordings, the slices were transferred to a recording chamber and perfused continuously at 2 mL/min with aCSF warmed to 30°C and bubbled with 100% O_2_. Slices were allowed to equilibrate for at least 30 min following transfer to the recording chamber and prior to recording.

### Electrophysiology

Magnocellular neurons in the SON and PVN were recorded under visual guidance using infrared illumination and differential interference contrast filters (IR‐DIC) on a fixed–stage upright microscope (Olympus BX51W). In slices from transgenic rats, MNCs were identified as GFP(+) VP neurons and GFP(‐), putative OT neurons in slices from VP‐GFP rats, or as RFP(+) OT neurons and RFP(‐), putative VP neurons in slices from OT‐RFP rats. VP‐GFP‐ and OT‐RFP–expressing neurons were located in slices using UV illumination and 488 nm/520 nm filters to detect GFP fluorescence and 595 nm/630 nm filters to detect RFP fluorescence. They were then targeted for whole–cell patch clamp recordings by switching to IR‐DIC. GABA–mediated IPSCs were recorded under voltage clamp at a membrane holding potential of 0 mV (i.e., the approximate reversal potential for glutamatergic synaptic currents) using a patch pipette solution containing (in mM): 110 D‐gluconic acid, 110 CsOH, 10 CsCl, 10 HEPES, 1 MgCl_2_, 1 CaCl_2_, 11 EGTA, 2 Mg‐ATP, 0.3 Na‐GTP. The pH was adjusted to 7.3 with KOH and the osmolarity to 300 mOsmol/l with D‐sorbitol.

Current–clamp recordings were performed using a patch pipette solution containing (in mM): 120 K‐gluconate, 10 KCl, 1 NaCl, 1 MgCl_2_, 1 CaCl_2_, 10 EGTA, 2 Mg‐ATP, 0.3 Na‐GTP, and 10 HEPES; the pH of the patch pipette solution was adjusted to 7.3 with KOH and the osmolarity was adjusted to 300 mOsmol/l with D‐sorbitol. The calculated liquid junction potential, 14 mV, is included in the resting membrane potential reported in the legend of Figure 5C. Patch pipettes were fabricated from borosilicate glass capillary tubes (1.65 mm outer diameter, 1.2 mm inner diameter; KG33; King Precision Glass) on a horizontal puller (P‐97, Sutter Instruments, Novato, CA). All recordings were performed with a Multiclamp 700B amplifier and a 1322A digitizer controlled by the pClamp Clampex program (Molecular Devices).

To use recorded bursts of IPSCs as templates for intracellular current injection, the continuous holding current was digitally subtracted from the template recording to yield a baseline of 0 pA, and the trace was multiplied by −1 to reverse the current direction (i.e., to produce a hyperpolarizing current template). The amplitude of the template file was scaled by 0.1, 0.2, 0.3…1 to produce the smallest effective amplitude that elicited a response in the different cells into which it was injected. The recordings used to generate the stimulus (i.e., the template recordings) were simultaneous paired recordings from two VP‐GFP (‐) neurons from a lactating rat that contained a pair of synchronized bursts flanked by several seconds of stochastic IPSC activity. Pairs of SON magnocellular neurons in slices from lactating rats that were selected for stimulation were GFP (‐), putative OT neurons that displayed rebound spiking following hyperpolarization (10–40 mV from threshold for 0.5–1 sec with intracellular “square” current pulses) (Stern and Armstrong 1995). Recordings of baseline (20 sec), stimulation (10 sec) and recovery (20 sec) were performed automatically by using sequencing keys in Clampex (Molecular Devices), such that the interruptions between these three sequential recording files were < 1 sec.

### Drugs and statistics

Tetrodotoxin (TTX, 1 *μ*mol/L) and NG‐Nitro‐L‐arginine methyl ester hydrochloride (L‐NAME, 100 *μ*mol/L) were purchased from Tocris (Ellisville, MO), aliquoted and stored at −20°C until use. TTX and L‐NAME were bath applied for 40 min. IPSCs were detected using the MiniAnalysis program (Synaptosoft). Measurements of IPSCs were taken from the last 30 min of drug application. The criteria used for IPSC burst classification were a burst duration ≥ 1.2 sec and an intra‐burst inter‐IPSC interval ≤ 200 msec (i.e., IPSC frequency ≥ 5 Hz). Bursts of IPSCs recorded simultaneously from pairs of neurons were considered synchronized if their onset times differed by ≤ 6 msec. We used the unpaired Student's *t*‐test for between–cell comparisons (Excel, Microsoft) if the data passed the D'Agostino‐Pearson test for normal (Gaussian) distribution (GraphPad Prism). We used the Mann‐Whitney test for between–cell comparisons (GraphPad Prism) for data that did not have a normal distribution. The chi square test was used to test for lactation–related differences in the number of cells exhibiting IPSC bursts. Data are presented as means ± SE. All probability (*P*) values are from one‐tailed tests as our hypothesis stipulated a lactation–related upregulation of IPSC bursts. All n values refer to numbers of cells.

## Results

**Figure 1:**
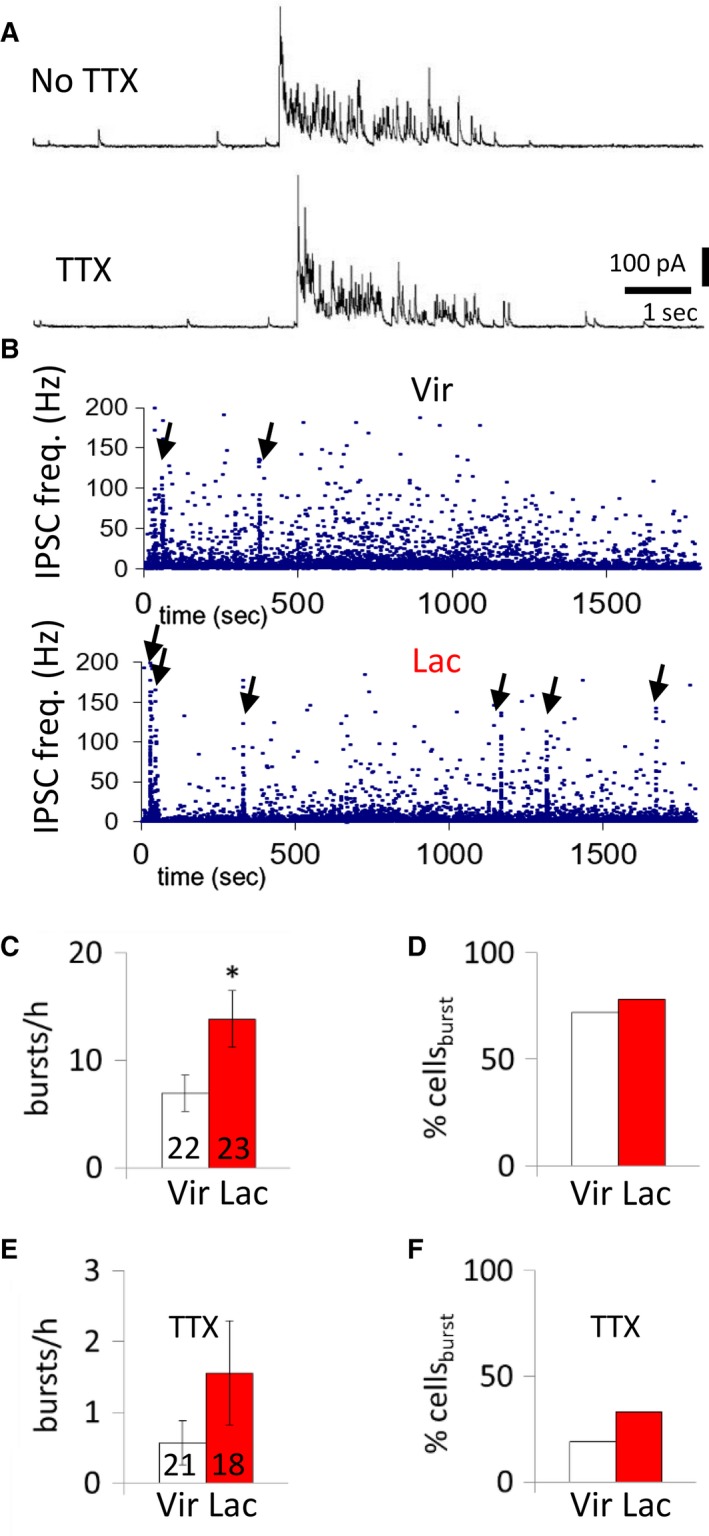
Effect of lactation on spontaneous IPSC bursts in MNCs. (A) Representative recording of bursts of IPSCs in an unidentified MNC from a lactating rat before and after the addition of TTX. (B) Time histogram of the instantaneous IPSC frequency recorded in representative MNCs. IPSC bursts are indicated by a sharp rise in instantaneous IPSC frequency; they occur with a higher incidence in the MNC from a lactating rat (arrows, lower) than the MNC from a non–lactating rat (arrows, upper). (C) Average incidence of bursts of IPSCs (bursts/h) in MNCs from virgin and lactating rats. (D) Percent of MNCs displaying IPSC bursts (% cells_burst_) in virgin and lactating rats. (E) Average incidence of bursts of IPSCs (bursts/h) in MNCs from non–lactating and lactating rats in the presence of TTX to block spiking. (F) Percent of MNCs displaying IPSC bursts in virgin and lactating rats in the presence of TTX. The numbers in the columns in all the figures represent the numbers of recorded cells in the averages. *, *P* <* *0.05.

We first recorded from MNCs in the OT neuron–rich dorsal region of the SON of wild–type virgin and lactating rats (Rhodes et al. 1981; Hou‐Yu et al. 1986; Gainer 2012). We recorded for 30 min in normal aCSF followed by a 30‐min recording period during which action potentials were blocked by the addition of 1‐*μ*mol/L TTX (Fig. 1A). While the percentage of MNCs that generated IPSC bursts was not increased with lactation (*P *=* *0.94, Chi square test), the average frequency of IPSC bursts recorded was increased in MNCs from lactating rats (nonlactating: 6.9 ± 1.6 bursts/h; lactating: 13.8 ± 2.6 bursts/h, *n* = 22, 23 neurons from 11, six rats, respectively; *P *=* *0.04, Mann‐Whitney test) (Fig. 1B–D). The IPSC burst duration (nonlactating: 2.5 ± 0.2 sec; lactating: 2.7 ± 0.2 sec, *n* = 16, 18 neurons, *P *=* *0.14, *t*‐test) and intra–burst IPSC frequency (nonlactating: 31.9 ± 3.1 Hz; lactating: 28.9 ± 2.4 Hz, *n* = 16, 18 neurons, *P *=* *0.11, *t*‐test) were not altered in MNCs from lactating rats. When action potentials were blocked with TTX, the percentage of OT neurons that generated IPSC bursts was not increased (*P* = 0.64, Chi square test), and, although a trend remained, the IPSC burst incidence was not significantly increased in the MNCs from lactating rats (nonlactating: 0.6 ± 0.3 bursts/h; lactating: 1.6 ± 0.7 bursts/h, *n* = 21, 18 neurons, *P *=* *0.10, Mann‐Whitney test) (Fig. 1E and F).

**Figure 2:**
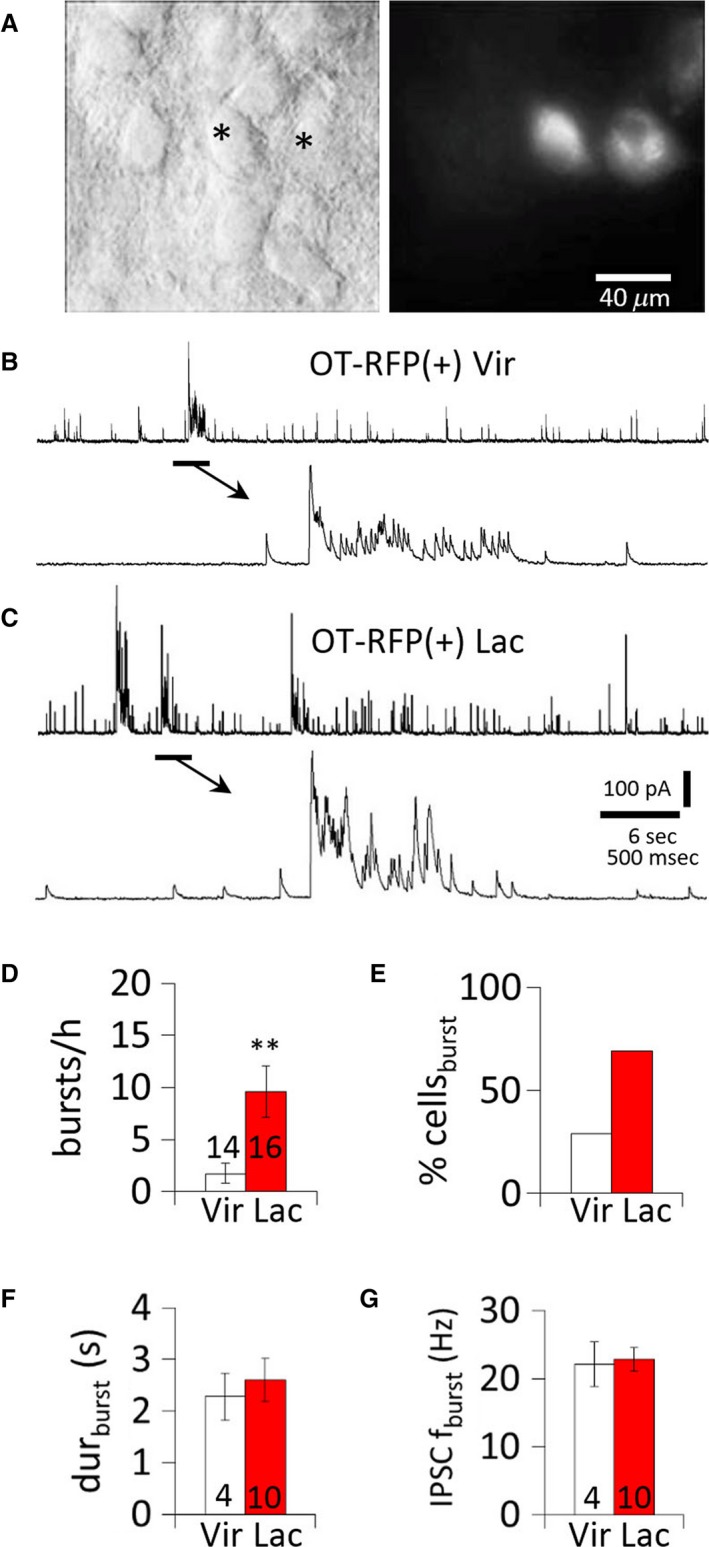
Upregulation of IPSC bursts in OT MNCs during lactation. (A) OT MNCs viewed in a brain slice under IR‐DIC (left) and epifluorescence (right). *, RFP(+) neurons. (B) Representative IPSC burst recorded in an OT RFP(+) MNC from a non–lactating rat. (C) Representative IPSC bursts recorded in an OT RFP(+) MNC from a lactating rat. (D) Average incidence of bursts of IPSCs (bursts/h) in OT‐RFP(+) MNCs from virgin and lactating rats. (E) Percent of OT‐RFP(+) MNCs displaying IPSC bursts (% cells_burst_) in virgin and lactating rats. (F) Mean IPSC burst duration in OT‐RFP (+) MNCs from virgin and lactating rats. (G) Mean IPSC frequency in IPSC bursts in OT‐RFP(+) MNCs from non–lactating and lactating rats. **, *P* <* *0.01.

We tested if the IPSC burst plasticity during lactation is specific to OT‐MNCs using transgenic OT‐RFP rats (kindly provided by Professor Yoichi Ueta, Katoh et al. 2011). Recordings of IPSCs in SON and PVN MNCs in slices from OT‐RFP rats revealed that the percentage of OT neurons that generated IPSC bursts was increased (*P *=* *0.006, chi square test) and that the frequency of IPSC bursts was higher in OT neurons from lactating rats compared to OT neurons from non–lactating rats (nonlactating: 1.7 ± 0.9 bursts/h; lactating 9.6 ± 2.4 bursts/h, *n* = 14, 16 neurons from five, four rats, respectively; *P* =* *0.007, Mann‐Whitney test) (Fig. 2A–E). There was no difference in the IPSC burst duration (nonlactating: 2.3 ± 0.5 sec, lactating: 2.6 ± 0.4; *P* =* *0.33, *t*‐test) or intra–burst IPSC frequency (nonlactating: 22.1 ± 3.2 Hz; lactating: 22. 9 ± 1.7 Hz, *n* = 4, 10 neurons from three, four rats, *P* =* *0.41, *t*‐test) in OT neurons from the two groups (Fig. 2B,C,F,G).

**Figure 3:**
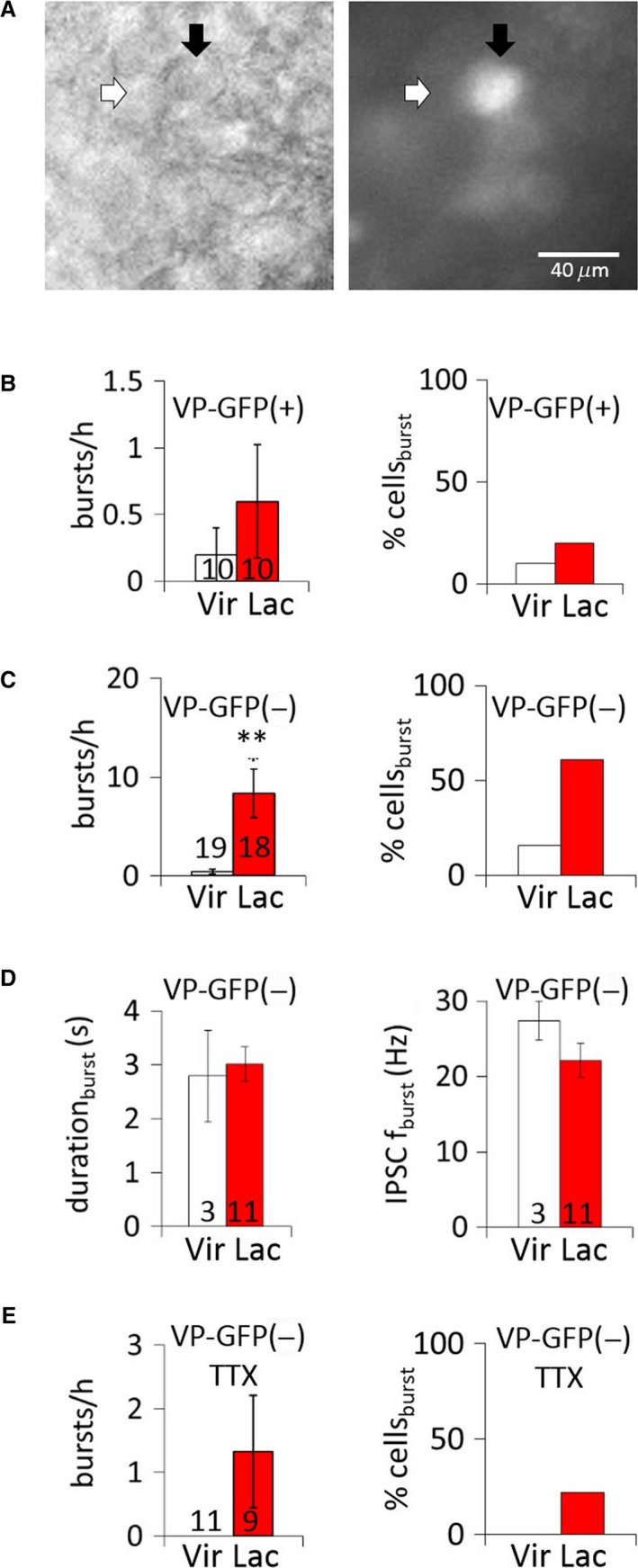
IPSC bursts in VP and putative OT MNCs during lactation. (A) Pair of MNCs viewed under IR‐DIC (left) and epifluorescence illumination (right). White arrow denotes a GFP(‐), putative OT neuron; black arrow denotes a GFP (+) VP neuron. (B) Average IPSC burst incidence (bursts/h) and percent of cells displaying bursts (% cells_burst_) in GFP(+) VP MNCs from virgin and lactating rats. (C) Average IPSC burst incidence (bursts/h) and percent of cells (% cells_burst_) displaying bursts in GFP(‐), putative OT MNCs from virgin and lactating rats. (D) Mean IPSC burst duration and intra‐burst IPSC frequency (IPSC f_burst_) in GFP(‐), putative OT MNCs from virgin and lactating rats. (E) Average IPSC burst incidence (burst/h) and percent of cells displaying bursts (% cells_burst_) in VP‐GFP(‐), putative OT‐MNCs from virgin and lactating rats in TTX. **, *P* <* *0.01.

We also performed recordings in SON MNCs in slices from VP‐GFP rats to test for changes in the IPSC bursts in VP‐MNCs. GFP(‐) neurons in these slices were considered putative OT‐MNCs (see Methods) (Fig. 3A) and served to assess IPSC burst plasticity in putative OT‐MNCs that were not subject to potential artifacts due to transgene expression. Neurons were recorded for 30 min in aCSF, followed by 30 min in 1‐*μ*mol/L TTX. The percentage of MNCs showing IPSC bursts and the IPSC burst incidence were not increased in VP neurons in slices from lactating rats (Fig. 3B). No IPSC bursts were detected in VP neurons recorded in the presence of TTX. Recordings from GFP(‐) OT‐MNCs revealed an increase in the percentage of MNCs showing IPSC bursts (*P* =* *0.0045, Chi square test) and an increase in the IPSC burst incidence during lactation (nonlactating: 0.4 ± 0.2 bursts/h; lactating: 8.3 ± 2.4 bursts/h, *n* = 19, 18 neurons from eight, seven rats, respectively; *P* =* *0.002, Mann‐Whitney test) (Fig. 3C). IPSC bursts in GFP(‐) OT‐MNCs from non–lactating and lactating rats were not different in duration (non‐lactating: 2.8 ± 0.8 sec; lactating: 3.0 ± 0.3 sec, *P* =* *0.38 *t*‐test) or IPSC frequency (nonlactating: 27.4 ± 2.6 Hz; lactating: 22.2 ± 2.2 Hz, *n* = 3, 11 neurons from three, three rats, respectively; *P* =* *0.14, *t*‐test) (Fig. 3D). During action potential blockade, the burst incidence in GFP(‐) OT‐MNCs was not significantly higher in lactating rats than in virgin rats (*P* =* *0.25) (Fig. 3E).

**Figure 4:**
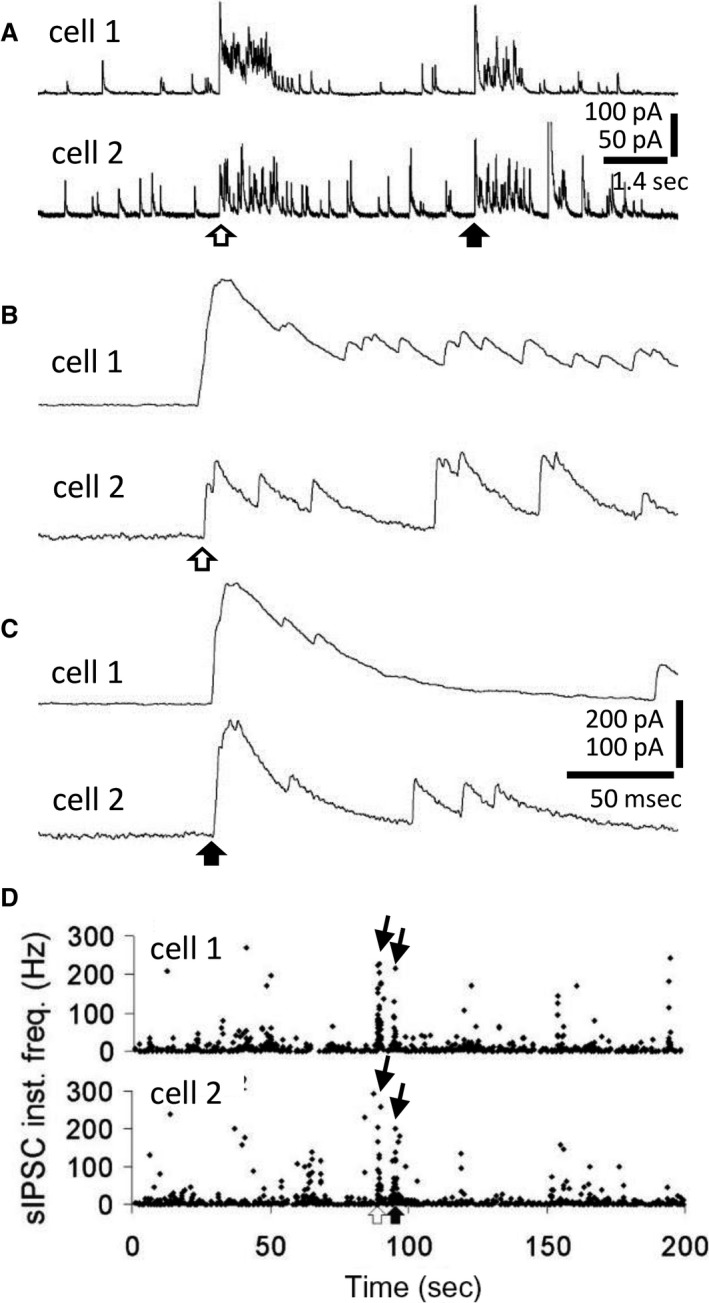
Onset–synchronized bursts of IPSCs in pairs of MNCs from lactating rats. (A) Paired recordings of onset–synchronized IPSC bursts (open and filled arrows) in two MNCs from a lactating rat. Some large IPSCs in cell 2 are truncated. (B, C) Expanded traces from A showing the onset synchronization of the IPSC bursts in each of the MNCs. Open and filled arrows correspond to the bursts in A. (D) Time histograms of the instantaneous frequency of IPSCs from the paired recording in A, illustrating the incidence (arrows) of the two synchronized bursts (white and black arrows below).

In our previous study, we found that a subset of IPSC bursts recorded in unidentified MNCs from male rats were sharply onset‐synchronized (Popescu et al. 2010). Synchronized GABAergic synaptic inputs to OT neurons have been predicted on the basis of “dual” or “shared” synapses. Since an increase in the number of shared synapses in lactating rats has been reported (Theodosis et al. 1981; Hatton and Tweedle 1982; Theodosis and Poulain 1984; Gies and Theodosis 1994, see also Perlmutter et al. 1984), we hypothesized that lactation would cause an increase in the incidence of synchronized IPSC bursts. Simultaneous whole–cell recordings of pairs of MNCs in the SON in slices from female rats revealed onset–synchronized bursts of IPSCs (Fig. 4). We found synchronized bursts in MNCs recorded in slices from lactating rats, but not in MNCs from non–lactating female rats. The mean time interval in the onset of synchronized IPSC bursts between cell pairs was 3.2 ± 0.9 msec; individual IPSCs within the bursts were not synchronized (Fig. 4B and C). In recordings from 26 pairs of MNCs from 13 lactating rats (six wild‐type, two VP‐GFP and five OT‐RFP rats), synchronized IPSC bursts were recorded in three pairs (11.5%). No synchronized IPSC bursts were recorded in 27 MNC pairs from 15 non–lactating rats (five wild type, five VP‐GFP and five OT‐RFP rats). One of the pairs of MNCs that showed synchronized IPSC bursts was a pair of VP‐GFP(‐), putative OT‐MNCs and the other two pairs were unidentified MNCs from wild–type rats. Thus, our data show that MNCs in lactating rats generate bursts of IPSCs that are synchronized between pairs of MNCs. When data were pooled from all lactating rats, the overall incidence of synchronized IPSC bursts was low, at 0.4 ± 0.2 bursts/h.

**Figure 5:**
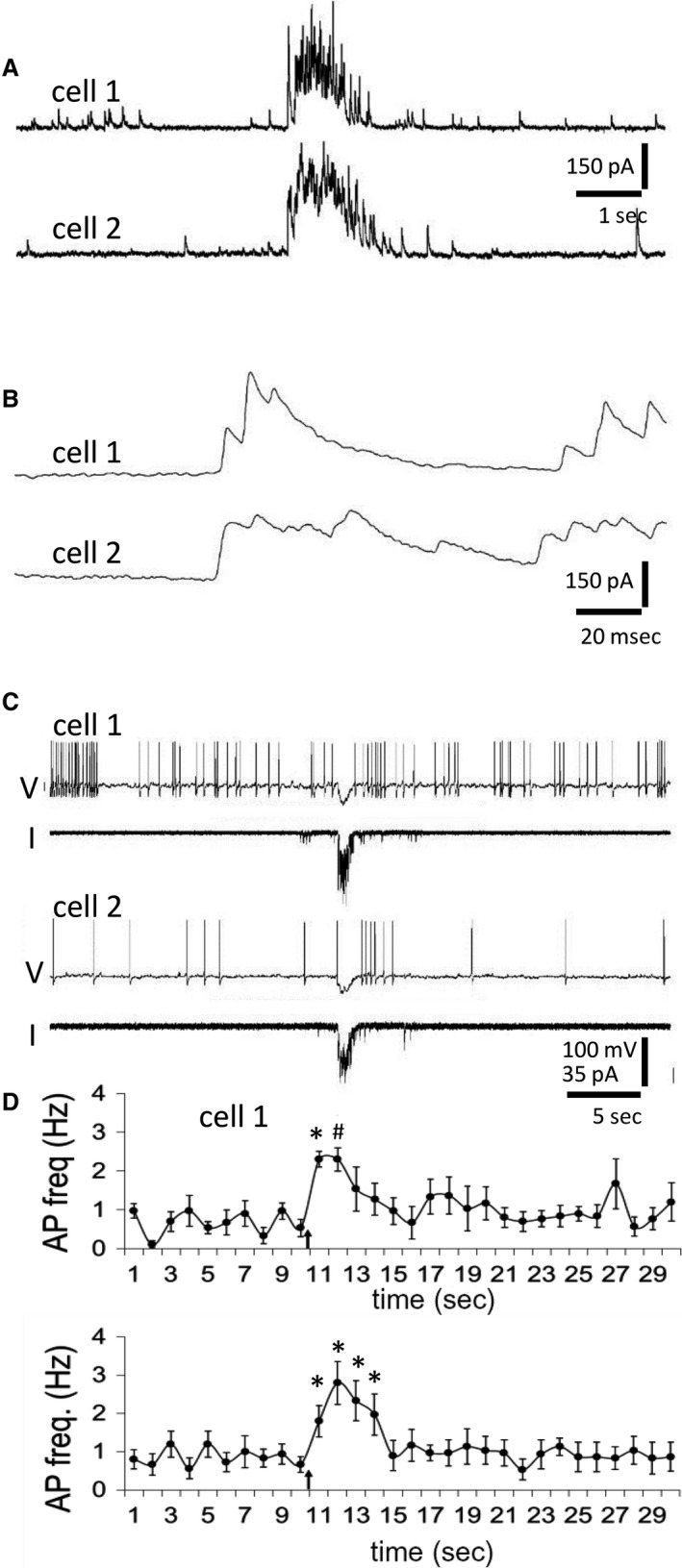
The effect of simulated synchronized IPSC bursts on action potential frequency in pairs of putative OT neurons. (A) Onset‐synchronized IPSC bursts recorded in a pair of VP‐GFP(‐), putative OT neurons from a lactating rat. (B) The onsets of the two bursts in A shown at an expanded time scale. (C) The IPSC bursts in A were used as templates for simultaneous intracellular current injections into two VP‐GFP(‐), putative OT neurons from a lactating rat recorded in current clamp. Shown are the membrane potential (V) and injection current (I) traces taken from two cells (cell 1 and cell 2) that received the IPSC burst current injections. The two cells responded to the simulated IPSC bursts with a hyperpolarization followed by a rebound increase in spiking frequency. Membrane potentials were held near threshold with current injection (Vm = −46 mV with + 30 pA of holding current in cell 1; Vm = ‐54 mV with + 10 pA of holding current in cell 2). (D) Time histograms of the mean spiking frequencies in MNC pairs (*n* = 5) before and after the synchronized IPSC burst current injections (arrows). The simulated IPSC bursts were followed by an increase in frequency in the cell pairs. *P <* *0.05, # *P* <* *0.01, repeated measures ANOVA and Dunnett's post hoc test.

While synchronized IPSCs have been recorded in pairs of OT‐MNCs from the supraoptic nucleus in organotypic cultures (Israel et al. 2003), onset–synchronized bursts of IPSCs are the only synchronized synaptic input recorded from MNCs in acute preparations. Because single IPSPs or square pulse hyperpolarizations in OT‐MNCs generate a rebound depolarization that can trigger spikes (Stern and Armstrong 1995, 1996; Israel et al. 2008), we sought to assess the effect of IPSC bursts on spiking in pairs of OT‐MNCs from lactating rats. This line of inquiry built on the finding that although the membrane potential dynamics of square pulses and single IPSPs are different from those of ramping–down IPSC bursts, burst–like currents can also elicit rebound spiking (Popescu et al. 2010). Importantly, we asked whether these endogenous, synchronized synaptic inputs can elicit in pairs of MNCs the emergence of trains of action potentials akin to those preceding milk ejection. Here, the effect of a single pair of bursts on spiking in pairs of MNCs was tested by injecting burst currents simultaneously into two MNCs. Recordings of an onset–synchronized pair of IPSC bursts obtained from a pair of GFP(‐), putative OT‐MNCs in a slice from a lactating rat (Fig. 5A and B) were stored and subsequently used as a template for current injections into pairs of putative OT‐MNCs, also from lactating rats (see Methods) (Fig. 5C and D). Pairs of neurons were chosen to be tested with IPSC burst current injection if, when held near threshold, they fired one or more action potentials immediately following a rectangular hyperpolarizing pulse (10–40 pA, 500 msec). Under these conditions, injection of the two onset–synchronized IPSC burst currents was followed by an increase in action potential frequency that overlapped temporally in the two MNCs (Fig. 5C and D).

**Figure 6:**
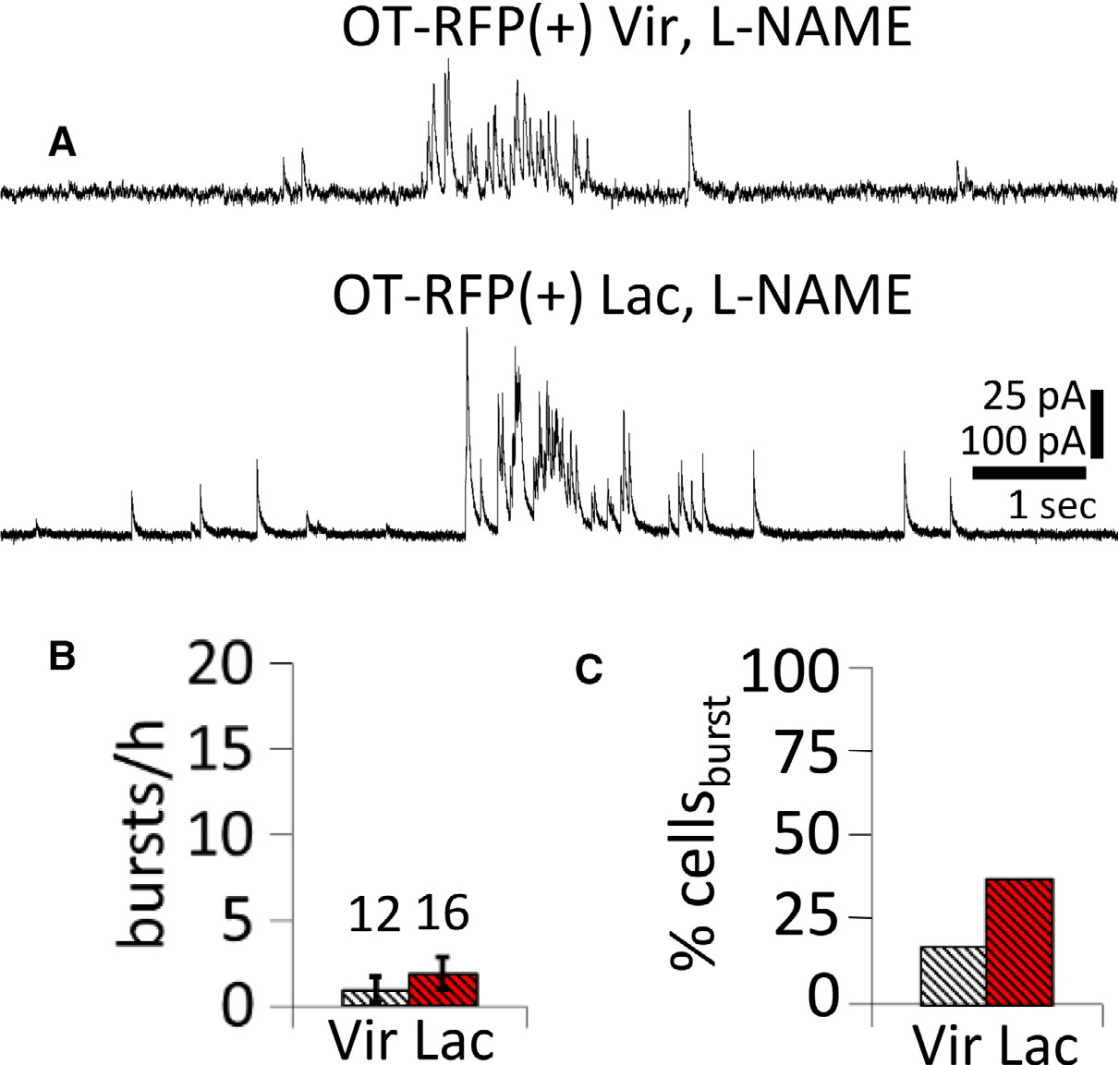
Nitric oxide dependence of lactation–induced increase in IPSC burst incidence. (A) Representative traces of IPSC bursts recorded in the NOS inhibitor L‐NAME (100 μmol/L) from a virgin rat (upper) and a lactating rat (lower). (B, C) Average incidence of IPSC bursts (burst/h) and percent of MNCs displaying IPSC bursts (% cells_burst_) recorded in the presence of 100‐μmol/L L‐NAME in slices from virgin and lactating rats. NOS inhibition abolished the increase in IPSC burst incidence in OT MNCs and the increased percentage of OT MNCs that generated IPSC bursts in slices from lactating rats.

Nitric oxide serves as a retrograde messenger that enhances GABA release onto MNCs in the PVN (Bains and Ferguson 1997; Stern and Ludwig 2001), and nitric oxide synthase (NOS) expression is upregulated in the PVN and the SON during lactation (Otukonyong et al. 2000). We therefore next tested for the nitric oxide dependence of the IPSC bursts by blocking NOS activity. RFP(+) OT neurons from non–lactating and lactating rats were recorded for 30 min in control aCSF, followed by 30 min in the broad–spectrum NOS inhibitor L‐NAME (100 *μ*mol/L). The percentage of cells displaying bursts and the incidence of bursts were no longer higher in lactating rats than in non–lactating rats following NOS inhibition (burst incidence: nonlactating: 0.9 ± 0.7 bursts/h, lactating: 1.9 ± 0.9 bursts/h, *P* = 0.19, Mann‐Whitney test; percentage of cells: *P* = 0.23, Chi square test; *n* = 12, 16 neurons from four, four rats, respectively) (Fig. 6).

## Discussion

The bursts of IPSCs reported here in slices from female rats and previously in slices from male rats (Popescu et al. 2010) demonstrate an abrupt, 10‐ to 50‐fold increase in the rate of release at GABA synapses on MNCs that lasts for hundreds of milliseconds. This capacity for generating long–lasting IPSC bursts persists in the absence of action potentials, although at a lower incidence (Popescu et al. 2010). Such a robust increase in inhibitory synaptic transmission is likely to have an impact on the post‐synaptic membrane potential by causing transient hyperpolarization followed by rebound depolarization (Armstrong et al. 1994; Ghamari‐Langroudi and Bourque 2000).

We observed onset–synchronized bursts of IPSCs in recordings from female rats. As in our recordings from male rats, the initial pair of IPSCs in synchronized bursts were sharply synchronized, but the subsequent individual IPSCs within the IPSC bursts were not. Since action potential‐evoked PSCs generated by collateral monosynaptic inputs would be expected to be synchronized, the lack of synchronization of individual IPSCs in the onset–synchronized bursts here is not consistent with action potential–mediated unitary IPSCs. This suggests that the bursts of sIPSCs in the hypothalamic MNCs are elicited by an action potential–independent exocytotic event. The reduction in the incidence of the IPSC bursts in TTX, however, suggests that action potentials facilitate IPSC burst generation.

The IPSC bursts may mediate a variety of functions. The fact that blockade of GABA_A_ receptors in the supraoptic nuclei suppresses the milk ejection reflex, and that IPSC bursts are onset‐synchronized in some pairs of MNCs, suggest that IPSCs may play a role in the synchronization of spike trains during the milk ejection reflex (Moos 1995; Voisin et al. 1995; Israel et al. 2008; Rossoni et al. 2008). Several studies have reported ultrastructural evidence for an increase during lactation in the number of GABA synapses shared between pairs of MNCs (Theodosis et al. 1981; Hatton and Tweedle 1982; Theodosis and Poulain 1984; Gies and Theodosis 1994; see also Perlmutter et al. 1984). The most parsimonious explanation for the generation of synchronized IPSC bursts in MNCs is that they originate at shared GABA synapses, perhaps due to a pre‐synaptic terminal microdomain increase in calcium, and their upregulation during lactation represents a potential mechanism for the coordination of MNC activity. Simulated IPSC burst currents injected into pairs of putative OT‐MNCs stimulated a coincident increase in spike frequency. While the simulated IPSC bursts did not trigger high–frequency spike trains, it is possible that neuromodulators present in vivo and absent in vitro may increase the excitability of the OT neurons, allowing them to spike at higher frequencies in response to rebound depolarization in vivo.

Our recordings revealed onset–synchronized IPSC bursts only in pairs of MNCs from lactating rats, albeit with a low incidence. We found onset–synchronized IPSC bursts in only 11% of recorded pairs of MNCs, only one of which was an identified OT neuron pair. Bursts of IPSCs occur infrequently (0.004 Hz in wild–type lactating rats), and “shared” synapses make up a small percentage of total GABAergic synapses (Theodosis et al. 1981; Perlmutter et al. 1984; Gies and Theodosis 1994). More paired recordings may make it possible to conclude definitively that lactation induces an upregulation of synchronized IPSC bursting. Nevertheless, we found that the frequency of IPSC bursts was increased several fold in MNCs from lactating rats, and that the upregulation was specific to OT neurons, which suggests a role for the bursts in lactation or lactation‐related behaviors. Interestingly, downregulation of IPSC bursts in MNCs was reported in a rat model of heart failure (Potapenko et al. 2011), suggesting that IPSC bursts may be regulated bidirectionally during different physiological states.

The OT neuron–specific upregulation of IPSC bursts in lactation is qualitatively similar to the increased number of GABA synapses on OT neurons, but not VP neurons, seen in ultrastructural studies (Gies and Theodosis 1994). However, the increase in the incidence of IPSC bursts in OT neurons (~ 6–20 ‐fold in OT‐RFP(+) and VP‐GFP(‐) MNCs) can be only partially explained by the ~ 30% increase in the number of GABAergic synapses. The increase in IPSC bursts may also depend on modulation of the release mechanism at GABAergic synapses, for example, via an increase in an extracellular neuromodulator, like nitric oxide, to increase the probability of IPSC burst generation.

The duration of IPSC bursts and the intra–burst IPSC frequency were not different in non–lactating and lactating rats. This indicates that the GABA release episodes responsible for the IPSC bursts, while more frequent during lactation, are identical once initiated in MNCs from lactating and non–lactating animals. What triggers the episodes of GABA release is unknown, but they are calcium‐dependent (Popescu et al. 2010). Since neuronal NOS expression is upregulated in the PVN and the SON during lactation (Otukonyong et al. 2000), and because NOS is activated by calcium (Abu‐Soud and Stuehr 1993), we tested whether nitric oxide plays a role in the upregulation of IPSC bursts in lactation. We found that during NOS blockade, IPSC bursts persisted, but their incidence was no longer higher in lactating rats than in non–lactating rats. Thus, whereas removal of calcium from the extracellular medium abolished the bursts of IPSCs entirely (Popescu et al. 2010), blocking NOS activity reduced their frequency, but did not abolish the IPSC bursts, which indicated that the calcium dependence of the IPSC bursts is independent of nitric oxide synthesis. Our data suggest that nitric oxide plays a role in the increased incidence of IPSC bursts during lactation, but that IPSC bursts can also be generated without significant involvement of nitric oxide.

The reversal potential of GABA_A_ currents (E_GABA_) becomes progressively more depolarized in lactating rats with successive litters, such that GABA IPSCs are inhibitory in most MNCs in primiparous dams and become excitatory in most MNCs by the third lactation (Lee et al. 2015). This constitutes an interesting mechanistic divergence of GABA function over iterations of the same behavior. The potentially changing role of IPSC bursts in controlling OT‐MNC activity will have to be tested in the context of a labile E_GABA_.

Milk ejection is thought to occur in all mammals, but to vary greatly in frequency from species to species (Capuco and Akers 2009). It would be instructive to compare the effect of lactation on IPSC bursts in brain slices from animals with different frequencies of milk ejection. For example in rats, milk ejection occurs every 3–10 min during an 18 h nursing period, whereas in rabbits, suckling is confined to a daily 3–5 min session containing 5–9 trains of action potentials (Paisley and Summerlee 1984). If the initiation of IPSC bursts is facilitated during lactation in order to support reflex milk ejection, then the IPSC bursts should be generated with higher frequency in lactating rats than in lactating rabbits.

In conclusion, we report a strong increase in bursts of IPSCs in oxytocinergic MNCs, but not vasopressinergic MNCs, during lactation. This reveals an upregulation of IPSC burst generation in female rats during lactation and suggests that IPSC bursts play a role in lactation–related, OT–mediated behaviors, including possibly a coordinating role in the milk ejection reflex.

## Conflict of Interest

None declared.
